# Embryonic ionizing radiation exposure results in expression alterations of genes associated with cardiovascular and neurological development, function, and disease and modified cardiovascular function in zebrafish

**DOI:** 10.3389/fgene.2014.00268

**Published:** 2014-08-07

**Authors:** Jennifer L. Freeman, Gregory J. Weber, Samuel M. Peterson, Linda H. Nie

**Affiliations:** School of Health Sciences, Purdue UniversityWest Lafayette, IN, USA

**Keywords:** cardiovascular, gene expression, heart rate, ionizing radiation, microarray, neurological, transcriptomics, zebrafish

## Abstract

The relationship between ionizing radiation (IR) and carcinogenesis is long established, but recently the association between IR and other diseases is starting to be recognized. Currently, there is limited information on the genetic mechanisms governing the role of IR in non-cancer related adverse health effects and in regards to an early developmental exposure. In this study, zebrafish embryos were exposed to a range of IR doses (0, 1, 2, 5, 10 Gy) at 26 h post fertilization (hpf). No significant increase in mortality or hatching rate was observed, but a significant decrease in total larval length, head length, and eye diameter was observed in the 10 Gy dose. Transcriptomic analysis was conducted at 120 hpf to compare gene expression profiles between the control and highest IR dose at which no significant differences were observed in morphological measurements (5 Gy). 253 genes with well-established function or orthology to human genes were significantly altered. Gene ontology and molecular network analysis revealed enrichment of genes associated with cardiovascular and neurological development, function, and disease. Expression of a subset of genetic targets with an emphasis on those associated with the cardiovascular system was assessed using Quantitative PCR (qPCR) to confirm altered expression at 5 Gy and then to investigate alterations at lower doses (1 and 2 Gy). Strong correlation between microarray and qPCR expression values was observed, but zebrafish exposed to 1 or 2 Gy resulted in a significant expression alteration in only one of these genes (*LIN7B*). Moreover, heart rate was analyzed through 120 hpf following IR dosing at 26 hpf. A significant decrease in heart rate was observed at 10 Gy, while a significant increase in heart rate was observed at 1, 2, and 5 Gy. Overall these findings indicate IR exposure at doses below those that induce gross morphological changes alters heart rate and expression of genes associated with cardiovascular and neurological functions.

## Introduction

Humans are continuously exposed to ionizing radiation (IR) through many sources. Two of the main exposures are related to background environmental sources and medical procedures. In the United States the average annual radiation dose received by the general public increased from 3.6 to 6.2 mSv since the 1980s mainly due to the significant increase of exposure to medical procedures (NCRP, 2009). The most common and well-studied health effect of IR is cancer, but recently the adverse health effects of IR exposure in other diseases are beginning to be realized. The strongest links to date include cardiovascular and nervous system diseases (Kim et al., 2008; Sanchez et al., 2009; McGale et al., 2011; Boerma, 2012). Data obtained from atomic bomb survivors, radiotherapy patients, and occupationally exposed radiation workers found an excess relative risk of circulatory disease ranging from 3 to 19% per Gy among different study populations (Little et al., 2010). Baker et al. (2011) examined the risk for developing cardiovascular disease from a wide range of radiation sources and suggested an association between cardiovascular disease and IR exposure not only at high doses, but also at low-to-moderate doses. In a recent review on the pathology, radiobiology, cardiology, radiation oncology, and epidemiology regarding radiation-related heart disease, the authors concluded that there is strong evidence of increased radiation-related heart disease with a longer latency period for lower dose levels (Darby et al., 2010). Studies also report a subtle effect on cognitive functioning in adolescents exposed to low-dose radiation *in utero* (Bar Joseph et al., 2004; Heiervang et al., 2010), although this effect is not shown in subjects who are exposed during adulthood (Yamada et al., 2002; Brummelman et al., 2011). Despite this strong evidence, there are major gaps in the current knowledge regarding a threshold dose of IR that induces cardiovascular disease and cognitive disorders. Current consensus in the literature indicates adverse cardiovascular effects are associated with IR doses greater than 2 Gy total body irradiation (Preston et al., 2003), while cardiovascular injury is observed at doses as low as 0.5 Gy (Shimizu et al., 2010). Less is known about the threshold dose related to neurological impacts and is being continually investigated (Mizumatsu et al., 2003; Achanta et al., 2007; Atwood et al., 2007).

The molecular and genetic mechanisms underlying the non-carcinogenic adverse health effects of IR are not well understood. Several mechanisms are suggested regarding radiation-related heart disease including macrovascular injury that accelerates age-related atherosclerosis leading to cardiovascular disease and microvascular injury that reduces capillary density leading to cardiovascular disease (Little et al., 2010). The pathways to these effects involve many biological responses including inflammation, antioxidative defense, endothelium signaling, and calcium regulation. In regards to the effects of IR on cognitive function, the proposed mechanisms include alteration of neuronal responses (Sanchez et al., 2009), modified antioxidant defenses (Di Toro et al., 2007), and other changes in specific molecular pathways (Verheyde and Benotmane, 2007). Overall a change in the normal and physiological levels of an organism to environmental stressors is ultimately a result of changes at the molecular and cellular levels. Moreover, the understanding of the immediate IR induced biological responses at the genetic level that trigger processes leading to short term and long term adverse health impacts such as those to the cardiovascular and neurological systems are not known. These genetic changes can be detected using transcriptomic technologies that permit thousands of genes to be analyzed in a single step to portray a genome-wide view of gene expression alterations. Few studies to date have used these technologies to identify transcriptomic signatures and genetic biomarkers related to IR exposure (Yin et al., 2003; Amundson et al., 2004; Dressman et al., 2007; Meadows et al., 2008, 2010; Lowe et al., 2009; Morandi et al., 2009; Kabacik et al., 2011; Wyrobek et al., 2011; Jaafar et al., 2013). These studies are starting to elucidate genetic targets associated with IR induced alterations, but were conducted in either cell culture systems, mature models, or from adult human peripheral blood samples. Thus, currently there is limited information on the genetic mechanisms of immediate and latent alterations associated with IR exposure during embryogenesis. Moreover, these studies are generally broad focused with minimal information regarding the association between IR exposure and cardiovascular or neurological alterations.

In this study, we first irradiated zebrafish embryos at different doses to establish IR toxicity and assess morphological alterations to establish baseline toxicity in our laboratory conditions following IR exposure at the specific developmental time point. The zebrafish is a complementary vertebrate model to assess the impacts of developmental exposures to environmental stressors. Zebrafish gained popularity in developmental and toxicological studies because of their rapid *ex utero* embryonic development that simplifies dosing (Hill et al., 2005). In addition, their near-transparent chorion permits visualization of morphological structures and internal organs including the brain, eyes, liver, kidney, and heart with a light microscope throughout the entire embryonic period. Moreover, a finished genome sequence and conserved genetic function between the zebrafish and human genomes permit translation of molecular mechanisms of toxicity observed in the zebrafish model system to humans (Barbazuk et al., 2000; Howe et al., 2013). Several zebrafish orthologs known to play key roles in human diseases are identified and mutations in these genes display phenotypes similar to those present in human diseases including cardiovascular and neurological alterations (Stainier et al., 1996; Dooley and Zon, 2000; Stainier, 2001; Incardona et al., 2004; Heideman et al., 2005; Peterson et al., 2011).

Furthermore, zebrafish may serve as a complementary and alternative model in light of current international discussions to reduce, refine, and replace animal bioassays (e.g., those discussed in Marone et al., 2014). Animal models are currently widely used in biological experiments. A dichotomy exists among these animal models. Many higher phylogenetic animals are used including mouse, rat, rabbit, canine, and non-human primates, mainly due to the fact that they display strong similarities to humans both in terms of their genetics and systems morphology. Animal models represent a significant burden not only financially, but also ethically in terms of the large number of higher order animals manipulated and sacrificed. On the other end of the spectrum, cell culture models serve as alternatives to replace animal model studies and have provided useful information, but are limited by the fact that only specific genetic pathways are applicable to human health. The zebrafish has the potential to satisfy both the need of an *in vivo* vertebrate model and to reduce the burden of mammalian animal bioassays. Thus, in this study, after establishing a dose at which no gross morphological alterations were observed, global gene expression analysis was performed using the zebrafish model system to identify altered genetic targets and pathways following the developmental IR exposure. The study of gene expression is an important aspect of understanding the genetic mechanisms associated with IR injury as differential gene expression plays a major role during critical periods of rapid cell growth in early development. Lastly, the functional implication of the immediate and later in development impacts of an embryonic IR exposure on heart rate was assessed to link identified genetic targets and functional consequences.

## Materials and methods

### Zebrafish husbandry

Adult zebrafish (*Danio rerio*) of the wild-type AB strain were housed on a 14:10 h light:dark cycle. Water temperature was maintained at 28°C with a pH of 7.2 and a conductivity range of 470–520 μ S. Fish were maintained and bred according to established protocols (Westerfield, 2007; Peterson et al., 2011). Fertilized embryos were collected at 1 h post fertilization (hpf), rinsed, sorted into individual wells of 96-well plates or into groups of 50 in petri dishes, and placed at 28°C until experimental use. Embryos were attained from multiple clutches for each experimental procedure. All animal protocols were approved and performed in accordance with Purdue University's Institutional Animal Care and Use Committee guidelines.

### Radiation dosing, toxicity, and morphological measurements at 1, 2, 5, and 10 Gy through 120 hpf

Zebrafish embryos in 96-well plates were irradiated at 26 hpf inside a Co-60 irradiator. All IR doses were given in full. Treatment groups of 1, 2, 5, or 10 Gy were irradiated for the appropriate length of time based on dose rate on day of dosing. The control groups were mock irradiated by placing the samples in the irradiator. Embryos were collected from different clutches on different days and irradiation repeated for each of these groups of embryos. Hatching rate and mortality were monitored daily throughout the developmental time course. At 120 hpf, morphological features were measured on multiple larvae from each treatment in each group using light microscopy on a Nikon SMZ1500 dissecting scope with NIS Elements imaging software (Melville, NY). Endpoints measured were eye diameter, head length, and total larval length (measured snout to tail). Morphological measurements were analyzed with an analysis of variance (ANOVA) and a *post-hoc* least significant difference (LSD) test (*p* < 0.05) with SAS software (version 9.2, SAS Institute Inc., Cary, NC) when a significant ANOVA was observed.

### Transcriptomic analysis at 5 Gy at 120 hpf

Transcriptomic microarray analysis was conducted to compare gene expression profiles between the control and highest IR dose at which no significant differences were observed in gross morphological measurements (5 Gy) following similar parameters as described previously (Peterson et al., 2011; Weber et al., 2013). Zebrafish were irradiated (5 Gy treatment) or mock irradiated (control treatment) at 26 hpf in groups of 50 in petri dishes (considered as subsamples). IR doses were given in full. At 120 hpf, larvae in each petri dish were pooled and homogenized in Trizol (Invitrogen, Carlsbad, CA) and flash frozen in liquid nitrogen. Three biological replicates (each consisting of 50 pooled larvae from separate clutches) were included. We have found in previous studies that pooling embryos and larvae aids in reducing biological variability allowing for a smaller sample size for microarray analysis (please see Peterson et al., 2011 and Weber et al., 2013 for examples of past studies in which this practice was successfully applied). Samples were stored at −80°C until RNA isolation was performed. Total RNA was isolated from larvae and converted to cDNA following established protocols (Peterson and Freeman, 2009a). Transcriptomic microarray analysis was conducted to compare gene expression profiles between the control and highest IR dose at which no significant differences were observed in gross morphological measurements (5 Gy) on the three biological replicates (*n* = 3). Microarray analysis was performed according to Peterson and Freeman (Peterson and Freeman, 2009b) with the zebrafish 385K expression platform (Roche NimbleGen, Madison, WI) using the one-color hybridization strategy. This platform contains 385,000 60-mer probes interrogating 37,157 targets with up to 12 probes per target. Following hybridization, arrays were washed and scanned at 5 microns using a GenePix 4000B array scanner (Molecular Devices, Sunnyvale, CA). Array image data was extracted using the NimbleScan software program (Roche NimbleGen, Madison, WI). Fluorescence signal intensities were normalized using quantile normalization (Bolstad et al., 2003) and gene calls generated using the Robust Multichip Average algorithm (Irizarry et al., 2003) following manufacturer recommendations.

Further statistical processing of the array data was performed with Array Star (DNASTAR, Inc., Madison, WI) and Ingenuity Pathway Analysis software (Ingenuity Systems, Redwood City, CA) to identify specific genes and molecular pathways altered following IR exposure. A robust and reproducible list of differentially expressed genes using recommendations from the Microarray Quality Consortium (Guo et al., 2006; Shi et al., 2006) was first determined by genes consistently expressed (Students *t*-test, *p* < 0.05) and substantially altered with a mean absolute log_2_ expression ratio of at least 0.585 (50% increase or decrease in expression). Genes identified to have significantly altered expression profiles were imported into Ingenuity Pathway Analysis for gene ontology and molecular pathway analysis following similar parameters as described in Peterson et al. (2011). All genes were converted and are reported as human homologs.

Quantitative PCR (qPCR) was performed on a subset of selected genes identified in the genomic microarray analysis with an emphasis on genes associated with cardiovascular development, function, and disease on the same samples used in the microarray analysis. Probes specific for target genes were designed using the Primer3 website (Rozen and Skaletsky, 2000). Target genes and associated primer sequences were *CACNA1D* (forward-AGGTCACCAAAGAGAAGACTGC, reverse-GAGGAAGATCGTCTTGACTGCT), *GUCY1B3* (forward-GAGAGAGAGGGTCTTCAGGACA, reverse-CCTCAAAACCATCCAAGTCTTC), *LIN7B* (forward-TGTTGTGTTCTTCTGTGGAACC, reverse-ATTTGCACTATCCAGCCAATTT), *NTRK3* (forward-AAGTCCTCTCCATCACGTCAAT, reverse- CTTGTTACAGTTGTGCCCATGT), *PBX3* (forward-ATCCTCTGTGATCTCCCCTACA, reverse- GTGGAAAAGTGGATGATTCCAT), and *PTPRE* (forward-ATAAACTCCACAACACGCACAC, reverse-GATTTCCGGTTCTCATGTTCTC). Similar to as performed in previous studies in our laboratory (e.g., Peterson et al., 2011, 2013; Zhang et al., 2011a; Weber et al., 2013; Wirbisky et al., 2014) several genes were assessed to determine the best reference gene to be used for this data set (data not shown). β-actin was found to be the most consistent and least variable for this analysis. The primer sequence for β-actin was the same as used in previous studies (e.g., Weber et al., 2013): forward-CTAAAAACTGGAACGGTGAAGG and reverse-AGGCAAATAAGTTTCGGAACAA. qPCR was performed on a CFX Connect™ Real-Time PCR Detection System (Biorad, Hercules, CA) using the iQ SYBR Green Supermix kit (Biorad, Hercules, CA) following similar methods as previously described (Peterson et al., 2011; Zhang et al., 2011a) following MIQE guidelines (Bustin et al., 2009). The cycling parameters included a 3 min. incubation phase at 95°C, 40 cycles of 95°C for 10 s, 60°C for 30 s, and 72°C for 30 s. Experimental samples were run in triplicate (technical replicates) and gene expression was normalized to β-actin. Efficiency and specificity were checked with melting and dilution curve analysis and no-template controls. Three biological replicates (*n* = 3) were analyzed and compared between the control and 5 Gy treatment to confirm gene expression alterations detected in the microarray analysis. An evaluation of linear correlation was performed and statistical significance of the correlation determined with a Pearson's correlation coefficient test using SAS software (*p* < 0.05) as described previously (Weber et al., 2013). For the correlation analysis, data input was log_2_ expression ratio for the microarray data and ratio of relative expression for the qPCR data for each gene.

### Gene expression analysis at 1 and 2 Gy at 120 hpf

To further investigate expression alterations in genes associated with cardiovascular development, function, and disease IR dosing was repeated at 0, 1, or 2 Gy at 26 hpf. All IR doses were given in full. Total RNA was isolated at 120 hpf from zebrafish larvae and converted to cDNA as described above. Data was analyzed with an ANOVA and a *post-hoc* LSD test (*p* < 0.05) with SAS software (version 9.2, SAS Institute Inc., Cary, NC) when a significant ANOVA was observed. Five biological replicates were included.

### Heart rate analysis at 1, 2, 5, and 10 Gy through 120 hpf

A baseline heart rate was measured visually with a light microscope at 24 hpf following a 15 min acclimation to room temperature (26°C). The number of heart beats was counted during a 10 s interval on all fertilized embryos and converted to heart beats per min (bpm). Zebrafish embryos were irradiated following measurement of baseline heart rate at 26 hpf as described above in 96-well plates. Embryos were collected from different clutches on different days and irradiation repeated for each of these groups of embryos. Treatment groups included irradiation at 1, 2, 5, and 10 Gy and a mock irradiated control group. All IR doses were given in full. The number of bpm was collected on the same individuals at multiple time points throughout development (26, 48, 72, and 120 hpf) as was performed to attain the baseline measurement and converted to bpm. All values are expressed as mean ± standard deviation. An ANOVA with a repeated measures test was used to analyze bpm among the different developmental time points with SAS software (*p* < 0.05).

## Results

### Toxicity and morphological alterations at 1, 2, 5, and 10 Gy through 120 hpf

**Figure 1:**
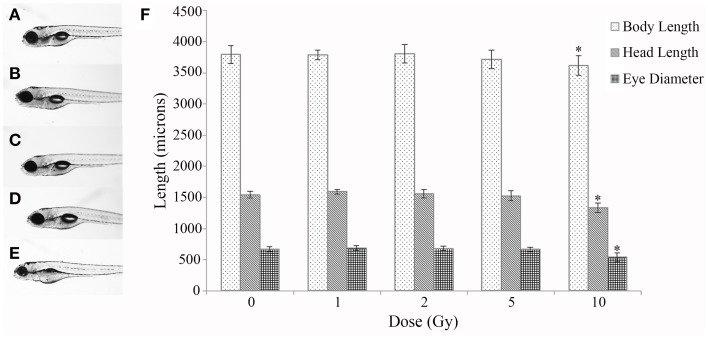
**Gross morphological measurements at 120 hpf**. Malformations and gross morphological measurements were assessed at 120 hpf in the control **(A)**, 1 Gy **(B)**, 2 Gy **(C)**, 5 Gy **(D)**, and 10 Gy **(E)** treatments. A significant decrease in body length, head length, and eye diameter was seen only in the 10 Gy treatment compared to the control treatment **(F)**. (*n* = 10–12; ^*^p < 0.05; error bars depict standard deviation).

A time course study at multiple IR doses was first conducted to determine toxicity and a threshold dose for gross morphological alterations. No significant difference was observed in mortality throughout the developmental time course with survival rates in all doses above 90% (Table 1). In addition, no significant difference was observed in hatching rate with over 95% of the individuals hatched by 72 hpf in all doses (Table 1). At 120 hpf gross morphological malformations were assessed and whole larval length, head length, and eye diameter were measured. A significant decrease in whole larval length, head length, and eye diameter was observed only in the 10 Gy dose (Figure 1) along with an increased frequency of general malformations including bent bodies and tails and pericardial edema.

**Table 1:** **Survival and hatching rate of zebrafish following developmental IR exposure**.

**IR dose (Gy)**	**Survival rate (%)**	**Hatching rate at 72 hpf (%)^a^**
0	93.8	97.8
1	97.9	97.9
2	100.0	95.7
5	95.6	100.0
10	92.2	100.0

a *Hatching rate is calculated only with surviving zebrafish*.

### Gene expression analysis at 5 Gy at 120 hpf

**Figure 2:**
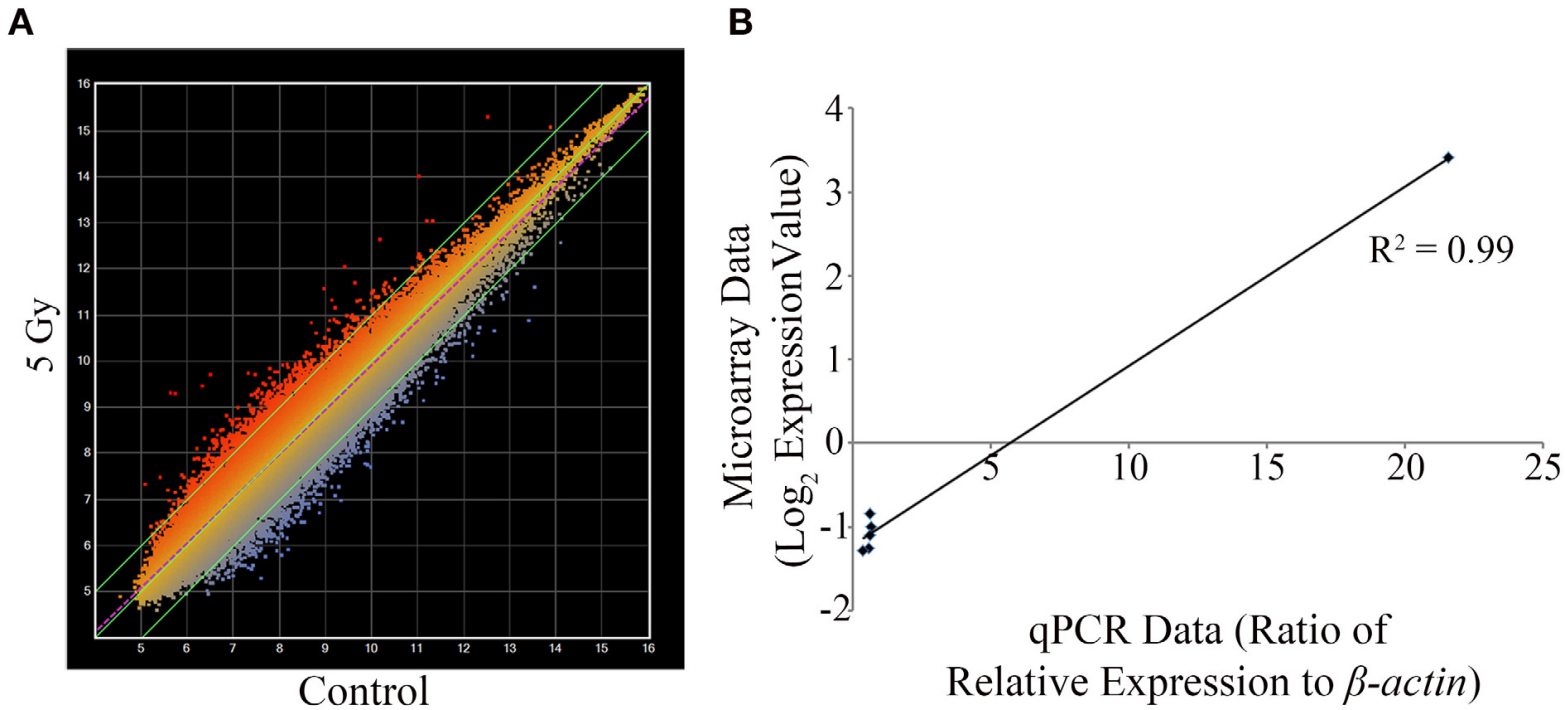
**Transcriptomic and qPCR comparative analysis at 5 Gy**. Mean intensity values for each probe are plotted with IR dosed larvae (y-axis) against control treatment (x-axis) **(A)**. Using set criteria 609 probes were altered at the 5 Gy dose. qPCR analysis was performed to compare gene expression changes detected on the microarray on six gene targets. A linear correlation was completed and statistical significance of the correlation determined using the Pearson's correlation coefficient test **(B)**. The data were statistically significant with a positive correlation between the microarray data and the qPCR data (*R*^2^ = 0.99, *R* = 0.995; *p* < 0.0001). The values for the microarray data are log_2_ expression values and for qPCR is the ratio of relative expression against β *-actin*. (*n* = 3).

From the overt toxicity assessment and the gross morphological measurements, transcriptomic microarray analysis was subsequently conducted to compare gene expression profiles of the highest IR dose at which no gross morphological alterations were observed (5 Gy) and the control treatment. Transcriptomic analysis performed at 120 hpf resulted in a scatter plot with an *R*^2^ correlation of 0.942 (Figure 2A). A total of 609 probes were altered with 65.5% of the probes down-regulated (399 probes) and 34.5% of the probes up-regulated (210 probes) (Supplementary Table 1). After accounting for redundant probes that targeted identical genes and removing probes targeting hypothetical proteins without substantial function or ontology information, annotation and pathway information was available for 253 genes orthologous to human genes with established functions. 65.2% of the genes (165 genes) were down-regulated, while 34.8% of the genes (88 genes) were up-regulated (Supplementary Table 2). Gene ontology analysis using Ingenuity Pathway Software classified genes into nonexclusive categories corresponding to cardiovascular disease, neurological and psychological disease, skeletal and muscle disorders, and genetic disorders as well as several molecular and cellular functions and physiological system development and function (Table 2). In addition, the top canonical pathways were synaptic long term depression (*p* = 1.35E-04), calcium signaling (*p* = 2.66E-04), CXCR4 signaling (*p* = 4.73E-04), HIF1α signaling (*p* = 7.16E-04), and synaptic long term potentiation (*p* = 1.05E-03). Genes with the greatest change in expression included *LIN7B* with an average log_2_ expression ratio of 3.510, *GPR24* with an average log_2_ expression ratio of 3.150, *RLN3* with an average log_2_ expression ratio of -2.263, and *GANC* with an average log_2_ expression ratio of −2.012.

**Table 2:** **Gene ontology of altered genes at 120 hpf in the 5 Gy treatment**.

**Biological function category**	***p*-value^a^**	**Number of genes^b^**
**DISEASES AND DISORDERS**		
Psychological disorders	3.43E-11—1.41E-02	64
Neurological disease	4.35E-11—1.41E-02	118
Skeletal and muscular disorders	3.61E-09—1.41E-02	95
Cardiovascular disease	3.51E-08—1.41E-02	77
Genetic disorders	6.22E-08—1.41E-02	161
**MOLECULAR AND CELLULAR FUNCTIONS**		
Cell-to-cell signaling and interaction	1.74E-07—1.41E-02	41
Cellular assembly and organization	2.71E-05—1.41E-02	40
Cellular function and maintenance	2.99E-05—1.41E-02	31
Small molecule biochemistry	2.99E-05—1.41E-02	41
Gene expression	1.98E-04—1.41E-02	7
**PHYSIOLOGICAL SYSTEM DEVELOPMENT AND DISEASE**		
Nervous system development and function	1.71E-05—1.41E-02	45
Tissue development	2.71E-05—1.41E-02	54
Behavior	4.02E-05—1.17E-03	27
Cardiovascular system development and function	4.65E-05—1.41E-02	23
Skeletal and muscular system development and function	1.69E-04—1.41E-02	18

a *Derived from the likelihood of observing the degree of enrichment in a gene set of a given size by chance alone*.

b *Classified as being differentially expressed that relate to the specified function category. A gene may be present in more than one category*.

Considering that one of the key enriched pathways in our genomic microarray analysis was cardiovascular disease and that recent studies indicate an association with IR exposure and cardiovascular disease (Darby et al., 2010; Little et al., 2010; Baker et al., 2011; McGale et al., 2011), a subset of genes with emphasis on those involved in cardiovascular development, function, and disease were chosen for confirmation of the microarray analysis using qPCR. Genes associated with cardiovascular development, function, or disease and chosen for qPCR analysis were *CACNA1D, GUCY1B3, NTRK3, PBX3*, and *PTPRE* (Table 3). In addition, *LIN7B* was also analyzed. The linear relationship between the microarray data and the qPCR relative expression ratio was analyzed using Pearson's correlation coefficient. A statistically significant positive correlation between these two data sets (*R*^2^ = 0.99, *R* = 0.995; *p* < 0.0001, Figure 2B) was observed confirming gene expression alterations.

**Table 3:** **Genes targeted for qPCR and their associated functions**.

**SEQID**	**Gene symbol**	**Function and/or association with cardiovascular development, function, and disease^a^**	**References**
OTTDART00000001972	*CACNA1D*	Significant role in sinoatrial and atrioventricular nodes function and in atrial fibrillation	Mancarella et al., 2008; Qu et al., 2011; Zhang et al., 2011b
ENSDART00000102452	*GUCY1B3*	Involved in development of atrioventricular canal and is a genetic variant associated with regulation of blood pressure and hypertension	Groneberg et al., 2010; Chang et al., 2011; Ehret et al., 2011
OTTDART00000024789	*LIN7B*	Neurological system	Jo et al., 1999; Sudo et al., 2006
ENSDART00000091728	*NTRK3*	Pathogenic copy number variant in congenital heart disease and multiple congenital anomalies, involved in heart development, and down regulated in cardiac hypertrophy	Srivastava and Olson, 1996; Dimberg et al., 1997; Lin et al., 2000; Kawaguchi-Manabe et al., 2007; Goldmuntz et al., 2011
ZV700S00006338	*PBX3*	Down regulation associated with cardiac defects representing developmental abnormalities affecting distinct stages of cardiac outflow tract development and corresponds to specific types of human congenital cardiac defects	Stankunas et al., 2008
OTTDART00000011315	*PTPRE*	Role in cardiac excitability	Tiran et al., 2003

a *LIN7B is not previously associated with cardiovascular development, function, or disease*.

### Gene expression analysis at 1 and 2 Gy at 120 hpf

**Figure 3:**
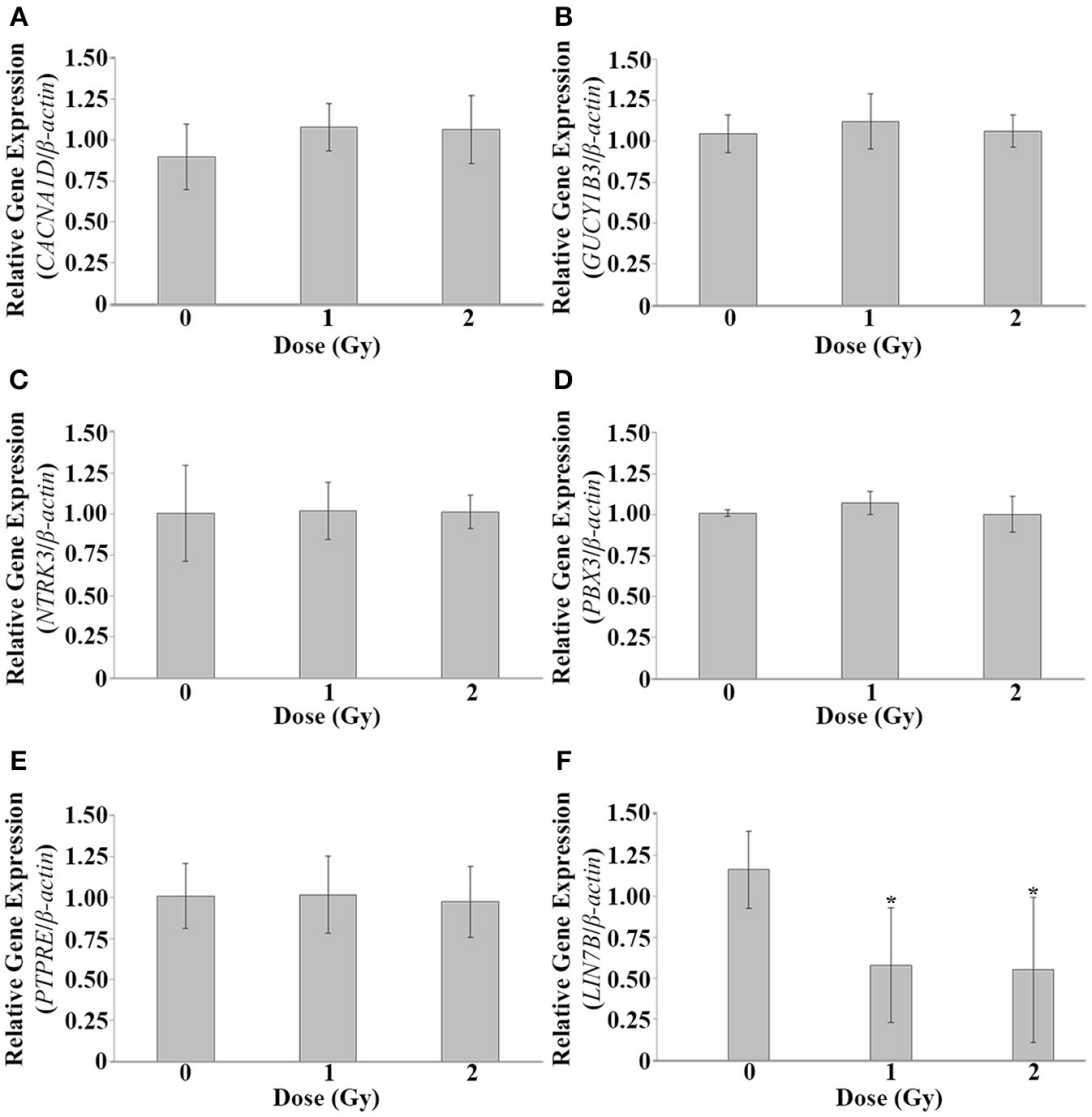
**Gene expression analysis at 1 and 2 Gy**. Gene expression alterations in *CACNA1D, GUCY1B3, LIN7B, NTRK3, PBX3*, and *PTPRE* were assessed at 120 hpf following dosing at 1 and 2 Gy at 26 hpf. No significant expression alterations were observed at these lower doses for *CACNA1D* **(A)**, *GUCY1B3* **(B)**, *NTRK3* **(C)**, *PBX3* **(D)**, or *PTPRE* **(E)**. A significant decrease in expression was observed at both 1 and 2 Gy for *LIN7B* **(F)**. (*n* = 5; ^*^p < 0.05; error bars depict standard deviation).

To investigate alterations of *CACNA1D, GUCY1B3, LIN7B, NTRK3, PBX3*, and *PTPRE* at lower IR doses, irradiation was repeated, and qPCR used to investigate gene expression alterations at 120 hpf at 1 and 2 Gy. No significant difference in gene expression was observed at these lower IR doses for *CACNA1D* (*p* = 0.1981), *GUCY1B3* (*p* = 0.4198), *NTRK3* (*p* = 0.9893), *PBX3* (*p* = 0.2534), or *PTPRE* (*p* = 0.9437), but a significant decrease in expression at both 1 and 2 Gy was observed for *LIN7B* (*p* = 0.0224) (Figure 3).

### Heart rate alterations at 1, 2, 5, and 10 Gy

**Figure 4:**
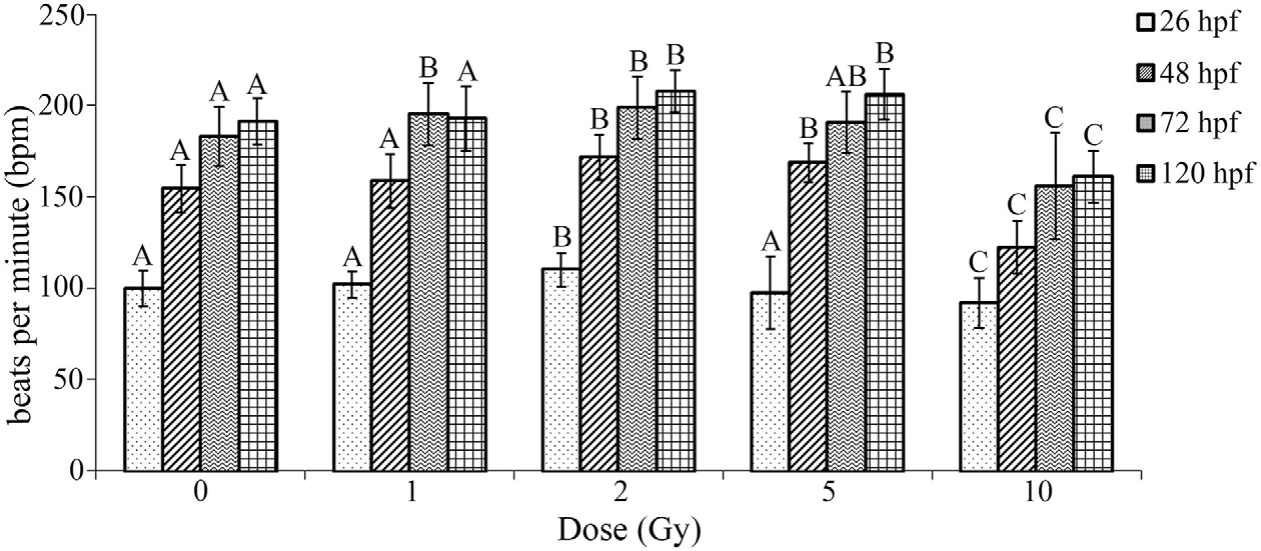
**Radiation induced alterations in heart rate in the zebrafish at 26, 48, 72, and 120 hpf**. At 26 hpf an increase in heart rate was observed at 2 Gy, while a decrease occurred at 10 Gy. At 48 hpf an increase in heart rate was observed at 2 and 5 Gy, while a decrease was seen at 10 Gy. At 72 hpf an increase in heart was observed at 1 and 2 Gy and the decrease in heart rate was still observed at 10 Gy. At 120 hpf an increase in heart was observed at 2 and 5 Gy, with a decrease in heart rate at 10 Gy. Overall during the developmental time course an increase in heart rate was observed at 1, 2, and 5 Gy, while a decrease in heart rate was seen at 10 Gy in comparison to the control. (*n* = 43–47; Different letters indicate significant differences compared to control at each time point at *p* < 0.05; error bars depict standard deviation).

To further investigate cardiovascular functional alterations associated with the IR doses used in our study, heart beat rate was measured at multiple time points throughout the developmental time course following IR exposure. During zebrafish development the initial peristaltic waves of contraction drive circulation and a heartbeat is visible by 24 hpf. Thus, a baseline heart rate measured as bpm was measured immediately preceding irradiation at 26 hpf. Heart rate was then measured in the same individual zebrafish at multiple time points throughout the developmental time course (26 hpf—immediately following irradiation, 48, 72, and 120 hpf). Similar to as reported in other studies, heart rate in our control treatment increased during development until reaching 180–200 bpm at 72 hpf (Glickman and Yelon, 2002; Incardona et al., 2004). Alterations in heart rate were dependent on dose with a significant increase in heart rate observed at 1, 2, and 5 Gy, while a significant decrease in heart rate was present in zebrafish irradiated at 10 Gy (Figure 4). Thus, irradiation at 26 hpf had significant and lasting alterations in heart rate.

## Discussion

IR is long established as a carcinogen with most studies focusing on DNA damage and mutations. Currently there are major knowledge gaps in characterizing the genetic and molecular mechanisms governing the relationship between IR exposure and diseases other than cancer. Recently, additional adverse health effects of IR were realized including alterations to the cardiovascular and nervous systems. Moreover, there is limited information on the immediate and latent effects of an embryonic exposure to these biological systems. To begin to elucidate IR toxicity in this study we first exposed embryonic zebrafish at 26 hpf to a range of IR doses to establish toxicity at doses that represent sublethal to potentially lethal whole body doses in humans. Zebrafish were observed through the developmental time course through 120 hpf and no significant increase in mortality or alterations in hatching rate was observed at any of the doses. In addition, an increase in malformations and a significant difference in morphology were only observed at 10 Gy. This initial toxicity assay provided baseline toxicity of IR in our laboratory at the specific doses at the specific developmental time point of exposure for comparison to previous completed studies with the zebrafish model system. Our findings are similar to those reported in the literature for radiation exposure at this developmental time point, while exposure at earlier developmental stages is reported as more toxic (McAleer et al., 2005; Geiger et al., 2006; Pereira et al., 2011). McAleer et al. (2005) and Geiger et al. (2006) observed that lethal effects of IR were inversely proportional to embryonic age with earlier embryonic stages (e.g., 2–6 hpf) being more sensitive to lethality than later embryonic stages (e.g., 8–24 hpf).

Previous studies using the zebrafish model system to evaluate ionizing radiation exposure have been used to assess general toxicity (e.g., mortality, hatching rates, gross morphological alterations, DNA damage, and apoptosis) (e.g., Bladen et al., 2007; Choi et al., 2010a,b, 2012a, 2013; Pereira et al., 2011; Sorrells et al., 2012; Yu et al., 2012; Toruno et al., 2014), to screen radiation protectors (McAleer et al., 2005; Geiger et al., 2006), and to assess the long-term effects on liver gene expression (Jaafar et al., 2013). To the best of our knowledge, no work is published on the effect of IR to heart rate using the zebrafish model. Heart rate is closely associated with cardiovascular function and the zebrafish near-transparent chorion permits visualization of internal organs including the heart during embryogenesis; hence, it is desirable to investigate how IR affects heart rate. During zebrafish development the initial peristaltic waves of contraction drive circulation and a heartbeat is visible by 24 hpf. Heart rate increases through the developmental time course until reaching 180–200 bpm between 48–72 hpf similar to what was observed in the fish in the control treatment (Glickman and Yelon, 2002; Incardona et al., 2004). At 120 hpf the heart has two chambers, the atrium and the ventricle. The atrium is located to the left and slightly posterior to the ventricle and the bulbus arteriosus is next the ventricle. Blood flows from the atrium to the ventricle and then through the bulbus ateriosus. Although the zebrafish heart is two-chambered compared to the four-chambered mammalian heart, the many processes of cell migration and differentiation involved in forming the zebrafish heart are parallel to those in mammalian heart development (Stainier, 2001). In this study an initial heart rate was attained at 24 hpf to attain a baseline heart rate prior to the IR exposure at 26 hpf. Heart rate analysis was conducted at multiple time points throughout the developmental time course and a significant decrease observed at 10 Gy, while a significant increase in heart rate was observed at 1, 2, and 5 Gy. While it can be concluded that the decrease in heart rate at 10 Gy is related to the gross malformations observed, the mechanism for the increase of heart rate at lower doses requires further investigation. Another potential mechanism for the increase and then decrease in heart rate could be explained by hormesis. Hormetic effects associated with exposure to alpha particles and microbeam photons were recently observed in zebrafish embryos (Choi et al., 2012a,b). Nonetheless, the increase in heart rate indicates a lasting cardiovascular impact of the developmental IR exposure and potential subtle alterations.

Zebrafish larvae at 120 hpf exposed to the highest dose of IR at which no significant difference was observed in malformations and morphology (5 Gy) were used for transcriptomic analysis to determine genetic targets and molecular pathways altered. Applying criteria from the Microarray Quality Control Consortium (Guo et al., 2006; Shi et al., 2006) a multi-tiered statistical processing approach was used and identified a differentially expressed gene list of 253 genes orthologous to human genes with established functions. As observed in past studies in our laboratory when evaluating transcriptome changes at doses below those that cause gross morphological malformations, gene expression changes were not of high magnitude (Peterson et al., 2011; Weber et al., 2013). As such to eliminate inflation of the false negative rate for this data set a Bonferroni correction test was not applied (please see Norris and Kahn, 2006 for a discussion of multiple correction tests not being applied in the analysis when gene expression changes are subtle and of smaller magnitude such as those observed in this study).

*LIN7B, GPR24, RLN3*, and *GANC* were the genes with the greatest magnitude of expression differences. *LIN7B* encodes a protein involved in protein binding and maintaining distribution of channels and receptors in the cell membrane and is involved in neurotransmitter secretion with previous association with schizophrenia. The protein encoded by *GPR24* is a member of the G protein-coupled receptor family 1 and is an integral plasma membrane protein which binds to melanin-concentrating hormone. The protein can inhibit cAMP accumulation and stimulate intracellular calcium flux. *RLN3* belongs to the family of relaxins that are endocrine and autocrine/paracrine hormones belonging to the insulin gene superfamily. In humans there are three non-allelic relaxin genes, *RLN1, RLN2*, and *RLN3*. While *RLN1* and *RLN2* share high sequence homology, *RLN3* does not. Relaxin has roles in the male and female reproductive system and also in regulating blood pressure, controlling heart rate, and releasing oxytocin and vasopressin. *GANC* encodes a glycosyl hydrolase enzyme that hydrolyzes the glycosidic bond between two or more carbohydrates or between a carbohydrate and non-carbohydrate moiety. This enzyme is key in glycogen metabolism and is associated with susceptibility to diabetes.

Few studies to date utilized transcriptomic technologies to investigate genetic mechanisms of IR response on non-cancer related health effects. These studies were conducted at a range of doses and in a variety of systems. All studies were conducted in either cell culture systems, in mature rodent models, or from adult human blood samples. Regardless, the differentially expressed gene list from this study was compared to each of these studies. There was minimal overlap between our differentially expressed gene list and those from the other studies. These differences are most likely due to the age of individuals, tissue analyzed, or in relation to dose response. There were similarities in up regulation of *CCNG1, MDM2*, and *RPS27L* and down regulation of *MAPK10* and *PAM* in previous studies with human lymphocytes and whole blood analysis (Fachin et al., 2007; Kabacik et al., 2011; Wyrobek et al., 2011). In addition, there was some overlap in genetic targets associated with cognitive function and neurological disease (e.g., *GRIA3* and *GRIN1*) in one study that analyzed 8–10 weeks male mice brain tissue 4 h after exposure to 0.1 or 2 Gy (Lowe et al., 2009). Furthermore, although there is limited overlap in the specific genetic targets, the top canonical pathways identified in our study and in the lower dose of 0.1 Gy are similar (i.e., synaptic long term depression and synaptic long term potentiation).

The dose of 5 Gy, albeit a higher dose of IR, was chosen for transcriptomic analysis as the highest dose for which no gross morphological measurements were observed and served as a starting point for this study for the assessment of gene expression alterations. To further investigate gene expression alterations, targeted expression analysis of five genes associated with cardiovascular functions and *LIN7B* was completed at 1 and 2 Gy. A significant decrease in expression of *LIN7B* was observed at both 1 and 2 Gy, while expression alterations were no longer observed for *CACNA1D, GUCY1B3, NTRK3, PBX3*, and *PTPRE*. Further work is needed to determine if overall enrichment of gene expression alteration categories are similar at the lower IR doses.

Overall this study indicates a developmental IR exposure at doses below those that induce gross morphological changes alters heart rate and expression of genes associated with cardiovascular and neurological development, function, and disease. This study furthers our understanding of the immediate IR induced biological responses during early development at the genetic level that trigger processes leading to short term cardiovascular effects. Future work is needed to define enrichment of gene expression alterations at the lower IR doses to provide additional information on transcriptome changes, to further investigate the functional impacts on the cardiovascular and nervous systems, and the lifespan consequences of the developmental IR exposure.

### Conflict of interest statement

The authors declare that the research was conducted in the absence of any commercial or financial relationships that could be construed as a potential conflict of interest.

## Abbreviations

ANOVA: analysis of variance
bpm: beats per minute
hpf: hours post fertilization
IR: ionizing radiation
LSD: least significant difference
qPCR: quantitative PCR.
