# Supplementary material for: Embryonic ionizing radiation exposure results in expression alterations of genes associated with cardiovascular and neurological development, function, and disease and modified cardiovascular function in zebrafish
Source: Front Genet. 2014 Aug 7;5:268. doi: 10.3389/fgene.2014.00268 (PMC4124797; doi:10.3389/fgene.2014.00268)
Supplement: Supplementary file 1 [file Presentation1.ZIP › Supp Table 2.PDF]

Supplementary Table 2. Genes altered at 120 hpf following irradiation at 5 Gy at 26 hpf.

| **Gene Name** | **SEQ_ID** | **$Log_2$ Expression Ratio** | **P-value** |
|---|---|---|---|
| *A2M* | ZV700S00004956 | -1.36854 | 0.0171 |
| *A2ML1* | TC247070 | -1.01073 | 0.00964 |
| *ABAT* | ZV700S00005573 | -0.95563 | 0.0332 |
| *ABCB1* | TC253259 | 0.95986 | 0.0342 |
| *ABCC8* | ENSDART00000073586 | -0.61084 | 0.0322 |
| *ABCF2* | ZV700S00006587 | -1.0287 | 0.0125 |
| *ABCG4* | OTTDART00000021604 | -0.79367 | 0.0367 |
| *ABHD3* | NM_001004569 | -0.73556 | 0.0343 |
| *ACBD5* | OTTDART00000028853 | -0.91204 | 0.0413 |
| *AFF2* | ENSDART00000074070 | -0.63573 | 0.0179 |
| *AKT3* | ENSDART00000092167 | -0.6032 | 0.00574 |
| *ALDH1L2* | ZV700S00002783 | -0.9924 | 0.0272 |
| *AMIGO1* | OTTDART00000013768 | -1.11147 | 0.012 |
| *ANK2* | ZV700S00002636 | -1.189 | 0.0305 |
| *APBA2* | ENSDART00000092406 | -0.623 | 0.045 |
| *AQP4* | OTTDART00000005078 | 1.121 | 0.0154 |
| *ARHGAP11A* | TC253885 | 0.96203 | 0.0273 |
| *ARHGEF9* | ZV700S00005847 | -1.71773 | 0.0487 |
| *ARHT1* | ZV700S00001490 | -1.47584 | 0.0249 |
| *ATP1A2* | OTTDART00000024863 | 0.96054 | 0.0121 |
| *ATP1A4* | TC239803 | -1.6361 | 0.00923 |
| *ATP5J* | ZV700S00003653 | 0.6438 | 0.036 |
| *BAZ2A* | ZV700S00004851 | -0.79854 | 0.0465 |
| *BCAP31* | OTTDART00000001758 | 0.65747 | 0.0154 |
| *BCMO1* | NM_200608 | 1.2866 | 0.0188 |
| *BIRC5* | ZV700S00001496 | 0.68934 | 0.0334 |
| *BIVM* | OTTDART00000024486 | -1.08777 | 0.0433 |
| *BRUNOL4* | OTTDART00000029266 | -1.13107 | 0.0358 |
| *C16orf44* | NM_001020580 | -0.6237 | 0.0269 |
| *C1GALT1* | OTTDART00000017572 | 0.93586 | 0.0342 |
| *C1orf63* | OTTDART00000029673 | -0.66617 | 0.0412 |
| *C20orf39* | OTTDART00000006460 | -1.1565 | 0.049 |
| *C3* | OTTDART00000023872 | -1.05893 | 0.00195 |
| *C3orf32* | OTTDART00000014521 | 0.7115 | 0.0296 |
| *C6orf125* | ZV700S00002641 | -1.59927 | 0.0406 |
| *CA10* | TC251476 | -1.10223 | 0.0493 |
| *CACNA1B* | OTTDART00000021541 | -0.60084 | 0.0222 |
| *CACNA1D* | OTTDART00000001972 | -0.86726 | 0.0298 |

| | | | |
|---|---|---|---|
| *CACNG8* | OTTDART00000020556 | -0.62593 | 0.016 |
| *CAPRIN2* | OTTDART00000009238 | 0.6617 | 0.0234 |
| *CCNF* | ZV700S00005353 | 0.87644 | 0.0466 |
| *CCNG1* | ZV700S00000580 | 0.67903 | 0.0171 |
| *CD109* | ENSDART00000085567 | 0.65547 | 0.00278 |
| *CHRNA3* | NM_001042684 | -0.63477 | 0.0116 |
| *CHRNE* | OTTDART00000022349 | 1.17784 | 0.0119 |
| *CHRNG* | ZV700S00001710 | 0.83193 | 0.0105 |
| *CHTF18* | ZV700S00003039 | -0.5904 | 0.0195 |
| *CIRBP* | OTTDART00000012149 | -0.91967 | 0.0139 |
| *CNFN* | OTTDART00000028225 | 0.77534 | 0.0485 |
| *COL8A1* | AI397462 | -1.34684 | 0.00586 |
| *COX6A2* | OTTDART00000027810 | 1.33047 | 0.0159 |
| *CPSF3* | OTTDART00000006434 | -0.63777 | 0.0233 |
| *CRYGB* | OTTDART00000027354 | 0.92503 | 0.0178 |
| *CSMD1* | ZV700S00002528 | -1.2282 | 0.0426 |
| *CTSS* | ZV700S00004121 | 1.02934 | 0.00618 |
| *CUTL2* | BI867269 | 0.84156 | 0.0337 |
| *CXCL12* | ZV700S00002779 | -1.55127 | 0.0255 |
| *CYGB* | ENSDART00000074114 | -1.12923 | 0.0411 |
| *CYP4V2* | NM_001077602 | -1.0881 | 0.0437 |
| *CYR61* | OTTDART00000023492 | 0.7046 | 0.00413 |
| *DAB2IP* | ZV700S00002086 | -1.63914 | 0.0398 |
| *DDC* | OTTDART00000026263 | -0.63154 | 0.00918 |
| *DES* | ZV700S00005046 | 0.64844 | 0.0427 |
| *DIDO1* | TC239513 | -0.97793 | 0.0367 |
| *DISP2* | OTTDART00000025626 | -1.18507 | 0.0404 |
| *DLG5* | AI721513 | -0.96627 | 0.00675 |
| *DMRT1* | OTTDART00000022546 | -1.00837 | 0.0207 |
| *DNAJB1* | ZV700S00004307 | 0.60714 | 0.0223 |
| *DOPEY2* | ENSDART00000078256 | -0.73607 | 0.0474 |
| *DTNB* | AY423026.1 | 0.69626 | 0.0419 |
| *ECHDC1* | OTTDART00000028197 | 1.57764 | 0.0246 |
| *ECM2* | OTTDART00000013676 | -0.73817 | 0.0136 |
| *EFCBP2* | OTTDART00000017757 | -1.13717 | 0.0238 |
| *EFNA5* | ZV700S00003078 | -0.8819 | 0.033 |
| *EIF4A1* | ZV700S00004747 | -0.7997 | 0.0469 |
| *ELA1* | OTTDART00000010719 | -0.69564 | 0.0419 |
| *ELAVL4* | ZV700S00005259 | -0.9701 | 0.0447 |
| *ELMO1* | TC250436 | -1.76654 | 0.0425 |
| *EN2* | ZV700S00005482 | -0.9911 | 0.0434 |

| | | | |
|---|---|---|---|
| *ENTPD5* | ENSDART00000103381 | 0.95053 | 0.0173 |
| *ERCC5* | NM_001014315 | 1.05934 | 0.0222 |
| *ERH* | NM_001020474 | -0.65674 | 0.0273 |
| *EVPL* | ENSDART00000082000 | -1.0866 | 0.0365 |
| *FAM57B* | BM316705 | -1.24927 | 0.0452 |
| *FGF17* | OTTDART00000025743 | 0.62754 | 0.0263 |
| *FNDC5* | OTTDART00000024987 | -0.7439 | 0.0447 |
| *FOSL2* | ZV700S00000052 | -0.8062 | 0.0422 |
| *FOXI1* | ZV700S00003468 | 0.7009 | 0.0304 |
| *FTH1* | OTTDART00000027842 | 1.2383 | 0.0113 |
| *GAD2* | TC251199 | -1.98417 | 0.0346 |
| *GALK2* | OTTDART00000028766 | 0.89866 | 0.0401 |
| *GANC* | AW419567 | -2.01243 | 0.0349 |
| *GFAP* | OTTDART00000024795 | 1.10363 | 0.00271 |
| *GNAZ* | ENSDART00000100865 | -1.01973 | 0.0493 |
| *GNE* | ZV700S00002387 | -1.48146 | 0.0202 |
| *GPR12* | OTTDART00000018851 | -0.87176 | 0.0325 |
| *GPR161* | OTTDART00000001380 | 0.6811 | 0.00151 |
| *GPR24* | OTTDART00000024568 | 3.15034 | 0.0000376 |
| *GPT* | ENSDART00000078033 | 1.1587 | 0.0441 |
| *GRAMD3* | ENSDART00000090534 | 0.7888 | 0.00469 |
| *GREM2* | ZV700S00003176 | 0.86534 | 0.0128 |
| *GRIA2* | ZV700S00002825 | -1.39703 | 0.0281 |
| *GRIA3* | NM_198360 | -1.197 | 0.0374 |
| *GRIN1* | ENSDART00000034849 | -1.0996 | 0.0353 |
| *GTPBP1* | BC056806.1 | 0.87336 | 0.0113 |
| *GUCY1B3* | ENSDART00000102452 | -1.0038 | 0.0148 |
| *HACE1* | ENSDART00000089855 | -0.9141 | 0.0366 |
| *HDAC1* | ZV700S00004348 | -1.18943 | 0.0299 |
| *HIF1A* | OTTDART00000026595 | -0.7548 | 0.0225 |
| *HK3* | ZV700S00000175 | 0.719 | 0.0488 |
| *HOOK1* | OTTDART00000005998 | -0.91374 | 0.00338 |
| *HOXA11* | TC263754 | 0.69284 | 0.00966 |
| *HOXA9* | ZV700S00003220 | 0.98777 | 0.0432 |
| *IGFN1* | NM_001014317 | 1.16497 | 0.0389 |
| *IGSF4D* | BC057517.1 | -1.1433 | 0.0403 |
| *IL6R* | ENSDART00000103256 | -0.65753 | 0.0418 |
| *ITIH3* | ZV700S00002489 | -0.8635 | 0.0423 |
| *ITPR3* | ZV700S00003930 | -0.9105 | 0.0429 |
| *KCNIP1* | OTTDART00000028396 | -1.10627 | 0.0491 |
| *KCNJ3* | OTTDART00000021895 | -0.5976 | 0.0147 |

| | | | |
|---|---|---|---|
| *KCTD13* | ZV700S00001150 | -1.42326 | 0.0497 |
| *KDR* | ZV700S00006270 | -0.94834 | 0.00842 |
| *LCAT* | ENSDART00000103791 | 0.77643 | 0.0468 |
| *LENG9* | ZV700S00000695 | 0.63583 | 0.0343 |
| *LIN7B* | OTTDART00000024789 | 3.58846 | 0.00369 |
| *LOX* | BC066465.1 | 0.85383 | 0.0477 |
| *LPHN3* | OTTDART00000014178 | -0.99294 | 0.0383 |
| *LPIN2* | OTTDART00000011687 | -0.61363 | 0.0234 |
| *LRP15* | BI709645 | -1.229 | 0.0448 |
| *LRRTM2* | ENSDART00000105477 | -1.21123 | 0.0412 |
| *LUZP2* | OTTDART00000030541 | -1.26146 | 0.048 |
| *LYPLA3* | BC066589.1 | 0.7435 | 0.0357 |
| *M17S2* | TC250619 | -0.84574 | 0.032 |
| *MAMDC1* | ENSDART00000033943 | -0.93257 | 0.0416 |
| *MAP2K5* | ENSDART00000052404 | -0.7164 | 0.044 |
| *MAP6* | TC242539 | -1.48267 | 0.032 |
| *MAPK10* | NM_001037701 | -0.9612 | 0.0421 |
| *MDM2* | OTTDART00000027215 | 0.79907 | 0.00703 |
| *MED11* | ZV700S00006025 | -0.73617 | 0.0371 |
| *MGC3123* | TC241341 | 1.49166 | 0.00363 |
| *MIA2* | OTTDART00000007261 | 0.814 | 0.0318 |
| *MIF4GD* | NM_001013284 | 0.64123 | 0.0376 |
| *MMP25* | TC256272 | 0.74877 | 0.0273 |
| *MPP2* | ZV700S00002781 | -1.65887 | 0.0419 |
| *MRAS* | OTTDART00000010901 | -1.1908 | 0.0431 |
| *MSP* | TC260416 | 0.63037 | 0.0432 |
| *MTHFD2* | ZV700S00005549 | 0.65467 | 0.0314 |
| *MTMR1* | OTTDART00000027753 | -0.76634 | 0.0425 |
| *MTMR12* | OTTDART00000022562 | -0.7614 | 0.0192 |
| *MYBPC1* | ZV700S00003536 | 0.80864 | 0.0118 |
| *MYBPC3* | OTTDART00000013552 | 0.8642 | 0.0487 |
| *MYCBP* | ZV700S00001871 | -0.59037 | 0.015 |
| *MYOM3* | ENSDART00000083574 | 0.8528 | 0.05 |
| *NAT2* | OTTDART00000015877 | -0.6101 | 0.0326 |
| *NCOA7* | ZV700S00003203 | 1.15347 | 0.0378 |
| *NGFB* | NM_199210 | 0.72526 | 0.037 |
| *NIF3L1* | OTTDART00000027738 | 0.68223 | 0.00607 |
| *NOD2* | ENSDART00000052380 | -0.7338 | 0.0336 |
| *NPHS2* | OTTDART00000011636 | -0.7927 | 0.00931 |
| *NTRK3* | ENSDART00000091728 | -1.19073 | 0.0263 |
| *OGFR* | OTTDART00000001830 | -0.62194 | 0.00877 |

| | | | |
|---|---|---|---|
| *OSGIN2* | ENSDART00000058973 | -1.49373 | 0.0127 |
| *OTOF* | OTTDART00000006081 | -0.77694 | 0.0302 |
| *P12757* | ENSDART00000031426 | -0.6537 | 0.0175 |
| *PAM* | ZV700S00002386 | -0.59047 | 0.0489 |
| *PANK1* | ZV700S00001261 | -1.1725 | 0.0393 |
| *PANX2* | OTTDART00000015055 | -0.82543 | 0.00361 |
| *PARP6* | TC242268 | -0.70996 | 0.0343 |
| *PBX3* | ZV700S00006338 | -1.28033 | 0.05 |
| *PCAF* | OTTDART00000020582 | -0.6835 | 0.0273 |
| *PCDHA7* | NM_001009586 | -1.1653 | 0.0419 |
| *PDE6C* | OTTDART00000025659 | -0.71183 | 0.0355 |
| *PDSS1* | TC259818 | 1.18557 | 0.0352 |
| *PFKM* | ZV700S00001077 | 1.1664 | 0.0223 |
| *PGM2* | OTTDART00000023819 | -0.62243 | 0.00394 |
| *PHLDA3* | ZV700S00000355 | 0.9205 | 0.0399 |
| *PMPCA* | ZV700S00004129 | 0.7752 | 0.0291 |
| *PPM1A* | OTTDART00000026160 | -0.61254 | 0.0169 |
| *PPP1R12C* | NM_001077579 | -0.8463 | 0.0207 |
| *PPP2R2C* | NM_001037394 | -1.06957 | 0.0361 |
| *PREB* | TC252253 | 1.00963 | 0.0208 |
| *PRG4* | OTTDART00000006801 | -0.91513 | 0.0388 |
| *PRKAG3* | TC263913 | 0.61887 | 0.0279 |
| *PRKAR1B* | NM_001076623 | -0.76413 | 0.0368 |
| *PRKCE* | TC253177 | -0.93287 | 0.0417 |
| *PRMT8* | OTTDART00000008711 | -0.656 | 0.0354 |
| *PRNP* | OTTDART00000025538 | -0.94566 | 0.0289 |
| *PRR12* | ZV700S00006606 | 1.51936 | 0.00397 |
| *PSMB8* | OTTDART00000011486 | 0.80057 | 0.0289 |
| *PTBP3* | ZV700S00005657 | -0.71823 | 0.0225 |
| *PTPRE* | OTTDART00000011315 | -1.1039 | 0.0413 |
| *PYGM* | NM_001020628 | 0.6843 | 0.0101 |
| *RALGPS2* | ENSDART00000098840 | -1.50417 | 0.048 |
| *RBMS3* | NM_001076716 | -0.596 | 0.0193 |
| *RHO* | OTTDART00000031060 | -0.64427 | 0.0441 |
| *RLN3* | OTTDART00000024306 | -2.2625 | 0.0404 |
| *RNF103* | BC081585.1 | -0.61974 | 0.016 |
| *RNPEP* | OTTDART00000028952 | 0.7797 | 0.0202 |
| *RPC8* | ZV700S00000938 | 0.60897 | 0.0485 |
| *RPE65* | ZV700S00000674 | -1.04703 | 0.0243 |
| *RPL13* | TC239904 | 0.6126 | 0.0326 |
| *RPS16* | TC257587 | 0.6902 | 0.0334 |

| | | | |
|---|---|---|---|
| *RPS27L* | ZV700S00006205 | 0.88734 | 0.0456 |
| *RTN4* | OTTDART00000024325 | 0.6105 | 0.0165 |
| *RUFY2* | ZV700S00003814 | -1.63843 | 0.0275 |
| *RUNDC3A* | ENSDART00000002393 | -1.26373 | 0.044 |
| *SACS* | ENSDART00000080251 | -1.03377 | 0.0425 |
| *SHOX2* | OTTDART00000029429 | -0.9931 | 0.0484 |
| *SLBP* | ZV700S00001073 | 0.69867 | 0.0481 |
| *SLC17A6* | NM_001009982 | -1.22184 | 0.0148 |
| *SLC24A2* | ZV700S00000328 | -0.75367 | 0.046 |
| *SLC24A3* | ENSDART00000048249 | -0.97753 | 0.0292 |
| *SLC24A4* | OTTDART00000029523 | -1.295 | 0.0188 |
| *SLC25A14* | OTTDART00000026697 | -0.94977 | 0.00758 |
| *SLC2A1* | OTTDART00000027037 | -1.347 | 0.0417 |
| *SLC35F2* | ZV700S00003527 | -0.75633 | 0.0407 |
| *SLC44A4* | OTTDART00000032117 | -1.10833 | 0.0293 |
| *SLC6A1* | ZV700S00004453 | -1.53236 | 0.0371 |
| *SLC6A15* | OTTDART00000030863 | -1.18103 | 0.0222 |
| *SMYD1* | NM_001039636 | 0.7899 | 0.0461 |
| *SNRK* | OTTDART00000028946 | -1.07687 | 0.0489 |
| *SPEG* | OTTDART00000024736 | -1.5485 | 0.0431 |
| *SPTBN1* | ZV700S00003416 | -0.66027 | 0.0456 |
| *SV2C* | OTTDART00000022585 | -0.6728 | 0.0235 |
| *SVIL* | ZV700S00004040 | -1.30117 | 0.00957 |
| *SYNGR1* | OTTDART00000024343 | -0.70787 | 0.0126 |
| *SYT7* | TC263189 | -1.4999 | 0.0435 |
| *TCAP* | ENSDART00000007293 | 0.60993 | 0.0499 |
| *TMEM170* | OTTDART00000015563 | -1.441 | 0.0398 |
| *TMEM181* | OTTDART00000008059 | 1.15683 | 0.0309 |
| *TMEM90A* | OTTDART00000005496 | -0.84873 | 0.0468 |
| *TMX2* | ZV700S00002576 | 0.86446 | 0.0407 |
| *TNRC4* | ENSDART00000105482 | -1.1834 | 0.0218 |
| *TOM1L2* | ENSDART00000097345 | 0.65634 | 0.0323 |
| *TRHDE* | OTTDART00000009401 | -1.10667 | 0.0245 |
| *TRIM9* | ZV700S00000557 | -0.8197 | 0.0498 |
| *TRIP13* | BC056713.1 | 0.6603 | 0.0268 |
| *TRPC1* | ENSDART00000087034 | -0.74084 | 0.0419 |
| *TSHR* | ENSDART00000054086 | 0.7397 | 0.05 |
| *TSHZ2* | ZV700S00002102 | -0.6393 | 0.0283 |
| *TTN* | OTTDART00000014487 | -0.81883 | 0.0371 |
| *UNC13A* | OTTDART00000011927 | -1.079 | 0.0204 |
| *USP28* | ENSDART00000003076 | 0.67924 | 0.0332 |

| | | | |
|---|---|---|---|
| *WLS* | OTTDART00000031307 | 0.61123 | 0.0466 |
| *WNT16* | OTTDART00000023277 | 0.73257 | 0.0253 |
| *WSCD1* | TC267124 | -1.14006 | 0.00906 |
| *XDH* | OTTDART00000011696 | -0.67927 | 0.0245 |
| *XKR4* | NM_001012258 | -1.14047 | 0.0469 |
| *XRCC4* | NM_200786 | 0.66026 | 0.0485 |
| *YARS* | OTTDART00000021451 | -0.6637 | 0.0313 |
| *ZBED4* | OTTDART00000012923 | -0.7997 | 0.00291 |
| *ZBTB22* | OTTDART00000002432 | -0.5947 | 0.0127 |
| *ZNF438* | NM_001077161 | -0.60287 | 0.0181 |
