# Supplementary material for: Embryonic ionizing radiation exposure results in expression alterations of genes associated with cardiovascular and neurological development, function, and disease and modified cardiovascular function in zebrafish
Source: Front Genet. 2014 Aug 7;5:268. doi: 10.3389/fgene.2014.00268 (PMC4124797; doi:10.3389/fgene.2014.00268)
Supplement: Supplementary file 1 [file Presentation1.ZIP › Supp Tables legends.pdf]

Supplementary Data

The data sets supporting the results of this article are included within the article and its additional files. Supplementary Table 1 contains all probes that were altered in the 5 Gy treatment analyzed by gene expression microarray at 120 hpf. Supplementary Table 2 contains all sequence IDs with their human homolog that was altered in the 5 Gy treatment analyzed by gene expression microarray at 120 hpf.
