# Supplementary material for: Embryonic ionizing radiation exposure results in expression alterations of genes associated with cardiovascular and neurological development, function, and disease and modified cardiovascular function in zebrafish
Source: Front Genet. 2014 Aug 7;5:268. doi: 10.3389/fgene.2014.00268 (PMC4124797; doi:10.3389/fgene.2014.00268)
Supplement: Supplementary file 1 [file Presentation1.ZIP › Supp Table 1.PDF]

Supplementary Table 1. Probes with altered expression at 120 hpf following irradiation at 5 Gy at 26 hpf.

**SEQ_ID**	**Gene**	**Log_2 Expression Ratio**	**P value**
AI397462	*COL8A1*	-1.347	0.0059
AI641363		-0.696	0.0154
AI721513	*DLG5*	-0.966	0.0068
AL916416		1.239	0.0171
AL925726		1.159	0.0336
AW019339		-0.895	0.0465
AW280155		-1.416	0.0356
AW419567	*GANC*	-2.012	0.0349
AW420546		-0.961	0.0365
AY423026.1	*DTNB*	0.696	0.0419
BC055245.1	*BC055245.1*	-0.857	0.0488
BC056713.1	*BC056713.1*	0.660	0.0268
BC056806.1	*GTPBP1*	0.873	0.0113
BC057517.1	*BC057517.1*	-1.143	0.0403
BC066465.1	*BC066465.1*	0.854	0.0477
BC066589.1	*LYPLA3*	0.744	0.0357
BC074098.1	*BC074098.1*	-1.275	0.0465
BC079485.1	*BC079485.1*	0.880	0.0174
BC081585.1	*BC081585.1*	-0.620	0.0160
BC081641.1	*BC081641.1*	-0.646	0.0459
BC081652.1	*PFKM*	1.003	0.0127
BC095696.1	*BC095696.1*	0.596	0.0023
BC097155.1	*BC097155.1*	-0.632	0.0438
BC151986.1		0.803	0.0052
BC152524.1		-1.156	0.0303
BI428775		-0.698	0.0214
BI563312	*Dr.83578*	-1.050	0.0084
BI709645	*Dr.86021*	-1.229	0.0448
BI867269	*CUTL2*	0.842	0.0337
BI878354		-0.705	0.0070
BI880705		-0.854	0.0070
BM072414		-1.019	0.0345
BM101616		-0.698	0.0299
BM316705	*BC007892*	-1.249	0.0452
BQ074552		-1.240	0.0431
CK705703		-2.319	0.0329
ENSDART00000002217		0.823	0.0467

ENSDART00000002393	*RUNDC3A*	-1.264	0.0440
ENSDART00000002540		-0.976	0.0037
ENSDART00000003076	*USP28*	0.679	0.0332
ENSDART00000003097		0.814	0.0345
ENSDART00000006357		-0.713	0.0232
ENSDART00000006376		-1.048	0.0281
ENSDART00000006476		0.715	0.0117
ENSDART00000006519		1.400	0.0491
ENSDART00000007293	*TCAP*	0.610	0.0499
ENSDART00000009653		-0.930	0.0249
ENSDART00000010026		-0.745	0.0249
ENSDART00000013229		-0.927	0.0278
ENSDART00000013830		-0.731	0.0419
ENSDART00000014210		-1.360	0.0124
ENSDART00000014395		0.719	0.0062
ENSDART00000015094		-0.826	0.0073
ENSDART00000015170		0.869	0.0471
ENSDART00000015848		-0.698	0.0454
ENSDART00000018144		-0.776	0.0271
ENSDART00000018441		-0.844	0.0270
ENSDART00000019504		-0.721	0.0366
ENSDART00000021944		-0.687	0.0287
ENSDART00000022393		0.869	0.0428
ENSDART00000023755		-0.665	0.0497
ENSDART00000026378		-0.627	0.0462
ENSDART00000026719	*Q5RGD1_DANRE*	-0.820	0.0338
ENSDART00000029774	*T55BB_DANRE*	-0.646	0.0131
ENSDART00000030938		-0.645	0.0054
ENSDART00000031426	*P12757*	-0.654	0.0175
ENSDART00000033943	*MAMDC1*	-0.933	0.0416
ENSDART00000034477		-0.720	0.0295
ENSDART00000034849	*GRIN1*	-1.100	0.0353
ENSDART00000036301		-0.679	0.0440
ENSDART00000039360		-0.688	0.0485
ENSDART00000040658		-0.793	0.0438
ENSDART00000040786		-0.967	0.0238
ENSDART00000041959		-1.664	0.0350
ENSDART00000043662		-0.986	0.0325
ENSDART00000044076		-0.889	0.0019
ENSDART00000044678		-0.889	0.0194
ENSDART00000047544	*CHRNE*	0.899	0.0214

ENSDART00000047816		-0.663	0.0046
ENSDART00000048249	*SLC24A3*	-0.978	0.0292
ENSDART00000052380	*NOD2*	-0.734	0.0336
ENSDART00000052404	*MAP2K5*	-0.716	0.0440
ENSDART00000053120		-0.716	0.0210
ENSDART00000053664		-0.852	0.0165
ENSDART00000054086	*TSHR*	0.740	0.0500
ENSDART00000054900		-1.317	0.0043
ENSDART00000056274		0.821	0.0104
ENSDART00000056671		-0.614	0.0341
ENSDART00000057211		-0.594	0.0444
ENSDART00000058973	*OSGIN2*	-1.494	0.0127
ENSDART00000059788		-0.623	0.0495
ENSDART00000059828		0.822	0.0454
ENSDART00000060324		-0.679	0.0381
ENSDART00000060666		0.871	0.0478
ENSDART00000061858		0.598	0.0408
ENSDART00000062445		1.227	0.0200
ENSDART00000062892		0.708	0.0386
ENSDART00000063200		-1.496	0.0473
ENSDART00000063264		0.615	0.0329
ENSDART00000063438		1.229	0.0082
ENSDART00000063537		-0.857	0.0285
ENSDART00000064672		-1.136	0.0478
ENSDART00000066043		-0.687	0.0206
ENSDART00000067294		1.112	0.0220
ENSDART00000067741		1.086	0.0291
ENSDART00000073411		-1.184	0.0393
ENSDART00000073450		1.050	0.0433
ENSDART00000073557		-1.232	0.0205
ENSDART00000073558		-1.202	0.0218
ENSDART00000073586	*ABCC8*	-0.611	0.0322
ENSDART00000074070	*AFF2*	-0.636	0.0179
ENSDART00000074103		-1.624	0.0364
ENSDART00000074114	*CYGB*	-1.129	0.0411
ENSDART00000074733		-0.736	0.0417
ENSDART00000075000		-1.214	0.0286
ENSDART00000075278		-0.756	0.0433
ENSDART00000075359		-1.202	0.0470
ENSDART00000075740	*Q561U3_DANRE*	-1.374	0.0284
ENSDART00000075790		-1.285	0.0314

ENSDART00000076077		0.648	0.0049
ENSDART00000076267		-0.758	0.0321
ENSDART00000076716		-1.002	0.0094
ENSDART00000076925		1.252	0.0292
ENSDART00000077056		0.931	0.0182
ENSDART00000077450		-0.982	0.0322
ENSDART00000077472		0.965	0.0287
ENSDART00000077929		-0.679	0.0236
ENSDART00000078033	*GPT*	1.159	0.0441
ENSDART00000078256	*Q9Y3R5*	-0.736	0.0474
ENSDART00000078376	*Q498V8_DANRE*	0.587	0.0441
ENSDART00000078554		-1.130	0.0326
ENSDART00000079176		-1.004	0.0330
ENSDART00000079219		0.871	0.0134
ENSDART00000079784		-1.073	0.0230
ENSDART00000079794		-0.963	0.0211
ENSDART00000080210		-0.697	0.0088
ENSDART00000080251	*SACS*	-1.034	0.0425
ENSDART00000080367		0.700	0.0329
ENSDART00000080477		0.665	0.0394
ENSDART00000080729		-0.762	0.0446
ENSDART00000080817		-2.299	0.0362
ENSDART00000081022		-1.807	0.0297
ENSDART00000081504		-2.272	0.0134
ENSDART00000082000	*EVPL*	-1.087	0.0365
ENSDART00000082114		0.692	0.0051
ENSDART00000082508		1.261	0.0205
ENSDART00000082956		-0.954	0.0252
ENSDART00000083266		0.602	0.0467
ENSDART00000083574	*MYOM3*	0.853	0.0500
ENSDART00000084252		1.001	0.0497
ENSDART00000084711		-0.834	0.0337
ENSDART00000084792		0.890	0.0103
ENSDART00000084879		-1.326	0.0499
ENSDART00000085218		-1.129	0.0487
ENSDART00000085567	*CD109*	0.655	0.0028
ENSDART00000085574	*CD109*	0.831	0.0316
ENSDART00000085661		-1.155	0.0406
ENSDART00000086317		-0.800	0.0341
ENSDART00000086492		-0.637	0.0488
ENSDART00000087034	*TRPC1*	-0.741	0.0419

ENSDART00000087084		-1.167	0.0458
ENSDART00000087570		-0.819	0.0122
ENSDART00000088408		-0.671	0.0029
ENSDART00000088588		-0.750	0.0226
ENSDART00000089855	*HACE1*	-0.914	0.0366
ENSDART00000090228		-1.077	0.0338
ENSDART00000090534	*GRAMD3*	0.789	0.0047
ENSDART00000090911		-0.818	0.0093
ENSDART00000091376		-1.190	0.0428
ENSDART00000091431		-0.957	0.0226
ENSDART00000091727	*NTRK3*	-1.489	0.0198
ENSDART00000091728	*NTRK3*	-1.191	0.0263
ENSDART00000091733	*NTRK3*	-1.105	0.0262
ENSDART00000091739		-0.655	0.0277
ENSDART00000091823		1.205	0.0226
ENSDART00000092007		0.791	0.0233
ENSDART00000092167	*AKT3*	-0.603	0.0057
ENSDART00000092264		-0.817	0.0089
ENSDART00000092406	*Q99767*	-0.623	0.0450
ENSDART00000092683		0.900	0.0392
ENSDART00000093312	*TNRC4*	-1.091	0.0217
ENSDART00000097110		-1.200	0.0331
ENSDART00000097282		-0.747	0.0479
ENSDART00000097308		-1.053	0.0494
ENSDART00000097345	*TOM1L2*	0.656	0.0323
ENSDART00000097367		-0.947	0.0114
ENSDART00000097488		-0.697	0.0157
ENSDART00000097608		-0.775	0.0262
ENSDART00000097678		0.674	0.0406
ENSDART00000097737		-0.840	0.0306
ENSDART00000097861		0.627	0.0395
ENSDART00000098117		-0.712	0.0008
ENSDART00000098213		-0.947	0.0434
ENSDART00000098697		0.613	0.0249
ENSDART00000098840	*NP_689876*	-1.504	0.0480
ENSDART00000099529		-0.721	0.0428
ENSDART00000099713		-0.848	0.0232
ENSDART00000099861		1.081	0.0478
ENSDART00000099934		-1.773	0.0333
ENSDART00000100250		-0.924	0.0329
ENSDART00000100685		-1.272	0.0304

ENSDART00000100865	*GNAZ*	-1.020	0.0493
ENSDART00000100918		1.493	0.0470
ENSDART00000101421		-1.478	0.0041
ENSDART00000101457	*MYOM3*	0.761	0.0403
ENSDART00000101780		-0.591	0.0375
ENSDART00000102039		0.600	0.0001
ENSDART00000102254		0.949	0.0448
ENSDART00000102342		-0.991	0.0362
ENSDART00000102452	*GUCY1B3*	-1.004	0.0148
ENSDART00000103138		-0.658	0.0264
ENSDART00000103256	*IL6R*	-0.658	0.0418
ENSDART00000103374		1.099	0.0152
ENSDART00000103381	*ENTPD5*	0.951	0.0173
ENSDART00000103791	*LCAT*	0.776	0.0468
ENSDART00000103889		1.089	0.0020
ENSDART00000104614		0.817	0.0433
ENSDART00000105477	*LRRTM2*	-1.211	0.0412
ENSDART00000105482	*TNRC4*	-1.183	0.0218
ENSDART00000105515	*Q58EF5_DANRE*	-0.610	0.0063
ENSDART00000105828		-0.764	0.0033
ENSDART00000106414		-0.757	0.0081
ENSDART00000106689		-1.176	0.0330
ENSDART00000106693		-0.619	0.0381
ENSDART00000106695		-0.702	0.0391
ENSDART00000106703		-1.340	0.0349
NM_001002537		-0.659	0.0347
NM_001003852		-0.916	0.0488
NM_001003884		0.793	0.0266
NM_001004569	*NM_001004569*	-0.736	0.0343
NM_001006033		0.639	0.0495
NM_001009586	*NM_001009586*	-1.165	0.0419
NM_001009594		-0.713	0.0487
NM_001009982	*NM_001009982*	-1.222	0.0148
NM_001012258	*XKR4*	-1.140	0.0469
NM_001013284	*MIF4GD*	0.641	0.0376
NM_001014315	*ERCC5*	1.059	0.0222
NM_001014317	*IGFN1*	1.165	0.0389
NM_001020474	*ERH*	-0.657	0.0273
NM_001020563		1.398	0.0299
NM_001020580	*NM_001020580*	-0.624	0.0269
NM_001020628	*NM_001020628*	0.684	0.0101

NM_001020687	*NM_001020687*	0.625	0.0449
NM_001024176		-0.690	0.0323
NM_001025451		0.601	0.0361
NM_001037394	*NM_001037394*	-1.070	0.0361
NM_001037701	*NM_001037701*	-0.961	0.0421
NM_001039636	*NM_001039636*	0.790	0.0461
NM_001039984		0.762	0.0033
NM_001040294		0.924	0.0142
NM_001042684	*NM_001042684*	-0.635	0.0116
NM_001044321	*NM_001044321*	0.718	0.0281
NM_001045353	*MGC153398*	0.681	0.0465
NM_001045357	*NM_001045357*	0.718	0.0374
NM_001045359		0.679	0.0199
NM_001076623	*NM_001076623*	-0.764	0.0368
NM_001076716	*NM_001076716*	-0.596	0.0193
NM_001077161	*NM_001077161*	-0.603	0.0181
NM_001077579	*NM_001077579*	-0.846	0.0207
NM_001077602	*NM_001077602*	-1.088	0.0437
NM_001077720		1.539	0.0366
NM_001077754	*NM_001077754*	-0.692	0.0321
NM_001079999	*NM_001079999*	-1.351	0.0437
NM_001080181		-1.195	0.0351
NM_001080606	*NM_001080606*	-0.716	0.0236
NM_001080659		0.861	0.0290
NM_001082955	*NM_001082955*	0.653	0.0264
NM_001083813	*NM_001083813*	-0.969	0.0297
NM_001083841	*NM_001083841*	-0.841	0.0014
NM_001089431		0.998	0.0075
NM_001089479	*NM_001089479*	-0.802	0.0201
NM_001098243		-0.938	0.0340
NM_001099243		0.977	0.0152
NM_130947		0.845	0.0126
NM_198360	*NM_198360*	-1.197	0.0374
NM_199210	*NM_199210*	0.725	0.0370
NM_200608	*NM_200608*	1.287	0.0188
NM_200786	*NM_200786*	0.660	0.0485
NM_205671		1.535	0.0361
NM_207093		1.120	0.0365
OTTDART00000001380	*GPR161*	0.681	0.0015
OTTDART00000001586		0.630	0.0277
OTTDART00000001758	*BCAP31*	0.657	0.0154

OTTDART00000001830	*OGFR*	-0.622	0.0088
OTTDART00000001972	*CACNA1D*	-0.867	0.0298
OTTDART00000002030		-1.163	0.0363
OTTDART00000002169		1.072	0.0474
OTTDART00000002229		-0.697	0.0148
OTTDART00000002284		-0.799	0.0215
OTTDART00000002332	*GRIN1*	-0.931	0.0396
OTTDART00000002432	*ZBTB22*	-0.595	0.0127
OTTDART00000004127		-0.653	0.0218
OTTDART00000005078	*AQP4*	1.121	0.0154
OTTDART00000005496	*TMEM90A*	-0.849	0.0468
OTTDART00000005889		-1.203	0.0398
OTTDART00000005997		-0.735	0.0449
OTTDART00000005998	*HOOK1*	-0.914	0.0034
OTTDART00000006075		0.774	0.0127
OTTDART00000006081	*OTOF*	-0.777	0.0302
OTTDART00000006434	*CPSF3*	-0.638	0.0233
OTTDART00000006460	*C20orf39*	-1.157	0.0490
OTTDART00000006501		-1.477	0.0371
OTTDART00000006801	*PRG4*	-0.915	0.0388
OTTDART00000006929		-0.739	0.0425
OTTDART00000007261	*MIA2*	0.814	0.0318
OTTDART00000007440		-0.647	0.0165
OTTDART00000007853		0.651	0.0236
OTTDART00000007879		0.631	0.0309
OTTDART00000008059	*TMEM181*	1.157	0.0309
OTTDART00000008087		0.722	0.0255
OTTDART00000008169		0.817	0.0065
OTTDART00000008711	*PRMT8*	-0.656	0.0354
OTTDART00000009024		-1.032	0.0425
OTTDART00000009238	*CAPRIN2*	0.662	0.0234
OTTDART00000009401	*TRHDE*	-1.107	0.0245
OTTDART00000010042	*AK097143*	0.587	0.0492
OTTDART00000010719	*ELA1*	-0.696	0.0419
OTTDART00000010901	*MRAS*	-1.191	0.0431
OTTDART00000011315	*PTPRE*	-1.104	0.0413
OTTDART00000011486	*PSMB8*	0.801	0.0289
OTTDART00000011636	*NPHS2*	-0.793	0.0093
OTTDART00000011687	*LPIN2*	-0.614	0.0234
OTTDART00000011691		-1.139	0.0386
OTTDART00000011696	*XDH*	-0.679	0.0245

OTTDART00000011927	*UNC13A*	-1.079	0.0204
OTTDART00000012149	*CIRBP*	-0.920	0.0139
OTTDART00000012923	*ZBED4*	-0.800	0.0029
OTTDART00000013002		1.191	0.0099
OTTDART00000013102		-1.003	0.0430
OTTDART00000013205	*C3*	-0.775	0.0263
OTTDART00000013552	*MYBPC3*	0.864	0.0487
OTTDART00000013553	*MYBPC3*	0.739	0.0187
OTTDART00000013676	*ECM2*	-0.738	0.0136
OTTDART00000013746		-0.841	0.0315
OTTDART00000013768	*AMIGO1*	-1.111	0.0120
OTTDART00000014178	*LPHN3*	-0.993	0.0383
OTTDART00000014372		-1.039	0.0348
OTTDART00000014487	*TTN*	-0.819	0.0371
OTTDART00000014521	*C3orf32*	0.712	0.0296
OTTDART00000015018		-0.728	0.0291
OTTDART00000015055	*PANX2*	-0.825	0.0036
OTTDART00000015092		-1.177	0.0277
OTTDART00000015563	*TMEM170*	-1.441	0.0398
OTTDART00000015725		-1.024	0.0224
OTTDART00000015877	*NAT2*	-0.610	0.0326
OTTDART00000016104		-0.667	0.0130
OTTDART00000016705		-1.128	0.0392
OTTDART00000017572	*C1GALT1*	0.936	0.0342
OTTDART00000017757	*EFCBP2*	-1.137	0.0238
OTTDART00000018346		-1.247	0.0465
OTTDART00000018463		-0.674	0.0189
OTTDART00000018496		-1.143	0.0389
OTTDART00000018851	*GPR12*	-0.872	0.0325
OTTDART00000020373		1.307	0.0276
OTTDART00000020556	*CACNG8*	-0.626	0.0160
OTTDART00000020582	*PCAF*	-0.684	0.0273
OTTDART00000020585		-0.625	0.0177
OTTDART00000021092		-1.016	0.0466
OTTDART00000021451	*YARS*	-0.664	0.0313
OTTDART00000021541	*CACNA1B*	-0.601	0.0222
OTTDART00000021604	*ABCG4*	-0.794	0.0367
OTTDART00000021803		-1.289	0.0287
OTTDART00000021804		-1.807	0.0437
OTTDART00000021838		-0.678	0.0217
OTTDART00000021895	*KCNJ3*	-0.598	0.0147

OTTDART00000022072		-1.189	0.0406
OTTDART00000022349	*CHRNE*	1.178	0.0119
OTTDART00000022546	*DMRT1*	-1.008	0.0207
OTTDART00000022562	*MTMR12*	-0.761	0.0192
OTTDART00000022585	*SV2C*	-0.673	0.0235
OTTDART00000022963	*Dr.79341*	0.792	0.0030
OTTDART00000023202		-1.194	0.0330
OTTDART00000023277	*WNT16*	0.733	0.0253
OTTDART00000023492	*CYR61*	0.705	0.0041
OTTDART00000023819	*PGM2*	-0.622	0.0039
OTTDART00000023872	*C3*	-1.059	0.0020
OTTDART00000024306	*RLN3*	-2.263	0.0404
OTTDART00000024325	*RTN4*	0.611	0.0165
OTTDART00000024343	*SYNGR1*	-0.708	0.0126
OTTDART00000024486	*BIVM*	-1.088	0.0433
OTTDART00000024568	*GPR24*	3.150	0.0000
OTTDART00000024578		0.993	0.0034
OTTDART00000024610		0.851	0.0346
OTTDART00000024643		0.915	0.0362
OTTDART00000024736	*SPEG*	-1.549	0.0431
OTTDART00000024788	*LIN7B*	3.233	0.0094
OTTDART00000024789	*LIN7B*	3.588	0.0037
OTTDART00000024795	*GFAP*	1.104	0.0027
OTTDART00000024863	*ATP1A2*	0.961	0.0121
OTTDART00000024987	*FNDC5*	-0.744	0.0447
OTTDART00000025384		0.820	0.0420
OTTDART00000025538	*PRNP*	-0.946	0.0289
OTTDART00000025578	*CACNA1D*	-0.826	0.0202
OTTDART00000025626	*DISP2*	-1.185	0.0404
OTTDART00000025659	*PDE6C*	-0.712	0.0355
OTTDART00000025743	*FGF17*	0.628	0.0263
OTTDART00000026160	*PPM1A*	-0.613	0.0169
OTTDART00000026196		1.040	0.0269
OTTDART00000026197		0.875	0.0418
OTTDART00000026263	*DDC*	-0.632	0.0092
OTTDART00000026595	*HIF1A*	-0.755	0.0225
OTTDART00000026697	*SLC25A14*	-0.950	0.0076
OTTDART00000026728		-0.781	0.0500
OTTDART00000026867		-0.861	0.0482
OTTDART00000027037	*SLC2A1*	-1.347	0.0417
OTTDART00000027090	*GAD2*	-1.448	0.0449

OTTDART00000027213	*MDM2*	0.851	0.0140
OTTDART00000027215	*MDM2*	0.799	0.0070
OTTDART00000027351		0.900	0.0284
OTTDART00000027353		0.862	0.0078
OTTDART00000027354	*CRYGB*	0.925	0.0178
OTTDART00000027370		0.628	0.0267
OTTDART00000027738	*NIF3L1*	0.682	0.0061
OTTDART00000027753	*MTMR1*	-0.766	0.0425
OTTDART00000027810	*COX6A2*	1.330	0.0159
OTTDART00000027842	*FTH1*	1.238	0.0113
OTTDART00000027999		-1.678	0.0130
OTTDART00000028078		-0.772	0.0119
OTTDART00000028197	*ECHDC1*	1.578	0.0246
OTTDART00000028225	*CNFN*	0.775	0.0485
OTTDART00000028266		0.749	0.0185
OTTDART00000028396	*KCNIP1*	-1.106	0.0491
OTTDART00000028621		-0.768	0.0002
OTTDART00000028766	*GALK2*	0.899	0.0401
OTTDART00000028853	*ACBD5*	-0.912	0.0413
OTTDART00000028868	*Dr.48047*	-1.030	0.0369
OTTDART00000028885		-0.742	0.0348
OTTDART00000028946	*SNRK*	-1.077	0.0489
OTTDART00000028952	*RNPEP*	0.780	0.0202
OTTDART00000029266	*BRUNOL4*	-1.131	0.0358
OTTDART00000029294		0.709	0.0309
OTTDART00000029429	*SHOX2*	-0.993	0.0484
OTTDART00000029523	*SLC24A4*	-1.295	0.0188
OTTDART00000029673	*C1orf63*	-0.666	0.0412
OTTDART00000030510		-1.048	0.0250
OTTDART00000030541	*LUZP2*	-1.261	0.0480
OTTDART00000030682		0.650	0.0341
OTTDART00000030695		-0.822	0.0381
OTTDART00000030775		-0.606	0.0474
OTTDART00000030863	*SLC6A15*	-1.181	0.0222
OTTDART00000031060	*RHO*	-0.644	0.0441
OTTDART00000031307	*AB097018*	0.611	0.0466
OTTDART00000031756		-0.786	0.0476
OTTDART00000032117	*SLC44A4*	-1.108	0.0293
OTTDART00000032447		0.696	0.0050
OTTDART00000032478		0.742	0.0420
OTTDART00000032685		-0.700	0.0346

TC235040		-0.820	0.0473
TC238133	*Dr.75351*	-0.838	0.0182
TC238755	*BAZ2A*	-0.664	0.0147
TC238927		2.436	0.0037
TC239408	*AY358744*	3.709	0.0022
TC239513	*DIDO1*	-0.978	0.0367
TC239696	*BC060857*	-0.746	0.0439
TC239803	*AK098076*	-1.636	0.0092
TC239904	*RPL13*	0.613	0.0326
TC239997		-2.048	0.0438
TC240635	*ATP5J*	0.734	0.0136
TC241341	*Dr.80915*	1.492	0.0036
TC241447		-1.103	0.0329
TC242038	*NCOA7*	1.059	0.0363
TC242268	*PARP6*	-0.710	0.0343
TC242539	*MAP6*	-1.483	0.0320
TC242812		-0.643	0.0148
TC244140		-1.144	0.0156
TC244237		-0.810	0.0481
TC244992		-1.980	0.0321
TC246153		-0.763	0.0425
TC246636		-1.296	0.0438
TC246970	*A2M*	-1.098	0.0022
TC247070	*AK123592*	-1.011	0.0096
TC249221		0.722	0.0122
TC250436	*ELMO1*	-1.767	0.0425
TC250619	*M17S2*	-0.846	0.0320
TC251199	*GAD2*	-1.984	0.0346
TC251476	*Dr.13585*	-1.102	0.0493
TC252253	*PREB*	1.010	0.0208
TC253177	*PRKCE*	-0.933	0.0417
TC253259	*ABCB1*	0.960	0.0342
TC253266		0.735	0.0377
TC253885	*ARHGAP11A*	0.962	0.0273
TC255102		-0.904	0.0179
TC256272	*MMP25*	0.749	0.0273
TC256493	*GPR24*	1.784	0.0074
TC257587	*RPS16*	0.690	0.0334
TC258033		-1.105	0.0132
TC258716		1.152	0.0323
TC259139		-1.372	0.0450

TC259196		0.765	0.0354
TC259493		0.637	0.0493
TC259818	*BC049211*	1.186	0.0352
TC260022		-1.468	0.0470
TC260416	*MSP*	0.630	0.0432
TC260674		0.788	0.0214
TC261494	*C3*	-0.986	0.0150
TC261727		0.734	0.0358
TC262455		-0.659	0.0214
TC262628		-1.273	0.0349
TC263189	*SYT7*	-1.500	0.0435
TC263716		-1.117	0.0138
TC263754	*HOXA11*	0.693	0.0097
TC263913	*PRKAG3*	0.619	0.0279
TC264976	*Dr.16758*	-0.649	0.0419
TC265218		-0.940	0.0464
TC266632		-0.677	0.0419
TC267124	*AB011095*	-1.140	0.0091
ZV700S00000052	*FOSL2*	-0.806	0.0422
ZV700S00000175	*HK3*	0.719	0.0488
ZV700S00000326		-1.299	0.0490
ZV700S00000328	*SLC24A2*	-0.754	0.0460
ZV700S00000355	*PHLDA3*	0.921	0.0399
ZV700S00000557	*AK126782*	-0.820	0.0498
ZV700S00000580	*CCNG1*	0.679	0.0171
ZV700S00000674	*RPE65*	-1.047	0.0243
ZV700S00000686	*PRKCE*	-0.783	0.0216
ZV700S00000695	*BC015921*	0.636	0.0343
ZV700S00000776		-1.475	0.0456
ZV700S00000938	*Dr.78164*	0.609	0.0485
ZV700S00000942		0.680	0.0455
ZV700S00001021		-1.165	0.0436
ZV700S00001022	*ELMO1*	-1.610	0.0415
ZV700S00001026		-0.672	0.0471
ZV700S00001073	*BT007433*	0.699	0.0481
ZV700S00001077	*PFKM*	1.166	0.0223
ZV700S00001150	*KCTD13*	-1.423	0.0497
ZV700S00001204		-1.520	0.0337
ZV700S00001261	*PANK1*	-1.173	0.0393
ZV700S00001483		-1.117	0.0393
ZV700S00001490	*ARHT1*	-1.476	0.0249

ZV700S00001496	*BIRC5*	0.689	0.0334
ZV700S00001572		-0.766	0.0493
ZV700S00001642		0.718	0.0186
ZV700S00001710	*CHRNG*	0.832	0.0105
ZV700S00001871	*MYCBP*	-0.590	0.0150
ZV700S00001995		-1.429	0.0498
ZV700S00002003		1.003	0.0289
ZV700S00002086	*DAB2IP*	-1.639	0.0398
ZV700S00002102	*BX640770*	-0.639	0.0283
ZV700S00002242		-0.592	0.0265
ZV700S00002386	*PAM*	-0.590	0.0489
ZV700S00002387	*GNE*	-1.481	0.0202
ZV700S00002480		-0.855	0.0482
ZV700S00002489	*ITIH3*	-0.864	0.0423
ZV700S00002528	*CSMD1*	-1.228	0.0426
ZV700S00002576	*TMX2*	0.864	0.0407
ZV700S00002636	*ANK2*	-1.189	0.0305
ZV700S00002641	*C6orf125*	-1.599	0.0406
ZV700S00002656		-0.731	0.0227
ZV700S00002779	*CXCL12*	-1.551	0.0255
ZV700S00002781	*MPP2*	-1.659	0.0419
ZV700S00002783	*AK095827*	-0.992	0.0272
ZV700S00002825	*GRIA2*	-1.397	0.0281
ZV700S00003039	*BC006437*	-0.590	0.0195
ZV700S00003078	*EFNA5*	-0.882	0.0330
ZV700S00003140		1.091	0.0296
ZV700S00003158		0.742	0.0192
ZV700S00003176	*GREM2*	0.865	0.0128
ZV700S00003200		1.019	0.0331
ZV700S00003203	*NCOA7*	1.153	0.0378
ZV700S00003220	*BT006990*	0.988	0.0432
ZV700S00003411	*Dr.77576*	0.592	0.0010
ZV700S00003416	*SPTBN1*	-0.660	0.0456
ZV700S00003468	*FOXI1*	0.701	0.0304
ZV700S00003527	*AK128062*	-0.756	0.0407
ZV700S00003536	*MYBPC1*	0.809	0.0118
ZV700S00003653	*ATP5J*	0.644	0.0360
ZV700S00003758		-1.539	0.0437
ZV700S00003781		-1.258	0.0366
ZV700S00003814	*Dr.117537*	-1.638	0.0275
ZV700S00003815	*SLC6A1*	-1.521	0.0395

ZV700S00003930	*ITPR3*	-0.911	0.0429
ZV700S00004002	*C3*	-0.719	0.0142
ZV700S00004040	*SVIL*	-1.301	0.0096
ZV700S00004047		-0.797	0.0328
ZV700S00004082	*ZNF207*	-1.392	0.0485
ZV700S00004121	*CTSS*	1.029	0.0062
ZV700S00004129	*PMPCA*	0.775	0.0291
ZV700S00004224		-1.502	0.0416
ZV700S00004307	*DNAJB1*	0.607	0.0223
ZV700S00004348	*Dr.31752*	-1.189	0.0299
ZV700S00004401		0.757	0.0128
ZV700S00004423	*A2M*	-0.749	0.0292
ZV700S00004453	*SLC6A1*	-1.532	0.0371
ZV700S00004525		-1.702	0.0328
ZV700S00004549		-0.672	0.0265
ZV700S00004576	*Dr.51201*	-0.684	0.0413
ZV700S00004714	*Dr.26174*	-1.295	0.0291
ZV700S00004747	*EIF4A1*	-0.800	0.0469
ZV700S00004771	*PANK1*	-1.142	0.0368
ZV700S00004851	*BAZ2A*	-0.799	0.0465
ZV700S00004956	*A2M*	-1.369	0.0171
ZV700S00005046	*DES*	0.648	0.0427
ZV700S00005168	*Dr.121787*	0.730	0.0208
ZV700S00005259	*ELAVL4*	-0.970	0.0447
ZV700S00005353	*CCNF*	0.876	0.0466
ZV700S00005482	*EN2*	-0.991	0.0434
ZV700S00005549	*MTHFD2*	0.655	0.0314
ZV700S00005573	*ABAT*	-0.956	0.0332
ZV700S00005657	*BC044585*	-0.718	0.0225
ZV700S00005847	*ARHGEF9*	-1.718	0.0487
ZV700S00005880		-1.491	0.0451
ZV700S00006025	*AF161414*	-0.736	0.0371
ZV700S00006156		0.694	0.0233
ZV700S00006205	*RPS27L*	0.887	0.0456
ZV700S00006270	*KDR*	-0.948	0.0084
ZV700S00006338	*PBX3*	-1.280	0.0500
ZV700S00006587	*ABCF2*	-1.029	0.0125
ZV700S00006606	*AB033031*	1.519	0.0040
ZV700S00006705		-0.879	0.0249
